# Regional Distribution of Causes of Death for Small Areas in Brazil, 1998–2017

**DOI:** 10.3389/fpubh.2021.601980

**Published:** 2021-04-27

**Authors:** Emerson Augusto Baptista, Bernardo Lanza Queiroz, Pedro Cisalpino Pinheiro

**Affiliations:** ^1^Asian Demographic Research Institute (ADRI), Shanghai University, Shanghai, China; ^2^Department of Demography, Universidade Federal de Minas Gerais (UFMG), Belo Horizonte, Brazil

**Keywords:** mortality, causes of death, ternary color coding, small-areas, micro-regions, Brazil

## Abstract

**Background:** What is the spatial pattern of mortality by cause and sex in Brazil? Even considering the main causes of death, such as neoplasms, cardiovascular diseases, external causes, respiratory diseases, and infectious diseases, there are still important debate regarding the spatial pattern of mortality by causes in Brazil. Evidence shows that there is an overlap in transitional health states, due to the persistence of infectious diseases (e.g., dengue, cholera, malaria, etc.,) in parallel with the increase in chronic degenerative diseases. The main objective of this paper is to analyze the spatio-temporal evolution of three groups of causes of death in Brazil across small areas from 1998 to 2017, by sex.

**Methods:** We use publicly available data from the System Data Mortality Information (SIM-DATASUS) from 1998 to 2017. We focus on this period due to the better quality of information, in addition to all deaths are registered following the Tenth Revision of the International Classification of Diseases (ICD-10). We estimate standardized mortality rates by sex and cause aggregated into three main groups. We use a ternary color scheme to maximize all the information in a three-dimensional array of compositional data.

**Results:** We find improvements in mortality from chronic degenerative diseases; faster declines are observed in the Southern regions of the country; but the persistence of high levels of mortality due to infectious diseases remained in the northern parts of the country. We also find impressive differences in external causes of deaths between males and females and an increase in mortality from these causes in the interior part of the country.

**Conclusions:** This study provides useful information for policy makers in establishing effective measures for the prevention of deaths and public health planning for deaths from external and non-communicable causes. We observed how the distribution of causes of death varies across regions and how the patterns of mortality also vary by gender.

## Introduction

In the last several decades, Brazil has experienced an accelerated decline in infant, child, and adult mortality (1). The median gain in life expectancy at birth between 1940 and 2015 was about 30 years (2). This change occurred much more quickly than any increase in life expectancy in developed countries over the same period. In 1940, life expectancy at birth in Brazil was around 45.5 years. It reached 67 years in 1990 and 75.5 years in 2015–2020. However, mortality rates observed around 2000 in several regions in the country were like observed rates in the US and Canada in the 1950s, thus showing that there is still room for improvement in terms of life expectancy in the region. In addition, there is wide variation in levels of mortality and life expectancy among and within Latin America countries (3, 4).

Followed by the rapid transition from high to low mortality, Brazil has also experienced a rapid epidemiological transition. However, this last process did not follow the same pattern shown in most industrialized countries and other Latin American countries, such as Chile, Cuba, and Costa Rica (5, 6). Empirical evidence shows that there is an overlap in transitional health states due to high levels of mortality from infectious diseases (e.g., dengue, cholera, malaria, etc.,) in parallel with the increase in mortality from chronic and degenerative diseases (6, 7). Thus, there is no linear path throughout all stages of the epidemiological transition, leaving it in the state of counter-transition (5, 6). In addition, morbidity and mortality are also at high levels, and can be characterized as going through a long-term process of transition and presenting clear regional variation over time. Moreover, there are contrasting epidemiological situations in different regions of the country, creating a scenario of epidemiological polarization (5–7).

However, less is known about the spatial pattern of mortality by cause and sex, even with respect to the main causes of death in Brazil, such as cardiovascular diseases (CVDs), neoplasms, external causes, respiratory diseases, and infectious diseases. Most studies on causes of death have been concentrated on epidemiological and public health aspects, as well as focused on the entire country, large geographic regions, states (federal units) or very specific municipalities (5, 6, 8–15). In summary, Brazil is divided in five major regions (North, Northeast, Southeast, South, and Center-West) comprising 26 states, plus the Federal District, and 5,565 municipalities (according to the 2010 census). These municipalities can be aggregated into 558 micro-regions, although, do not constitute political or administrative entities. Therefore, there is an absence of and a need for studies on variation in mortality over time and space, especially for small areas, although, over the past years there has been an increasing interest in identifying regional differences in mortality within the country for smaller areas (16–19). Policies for public health and well-being must be spatially focused to serve an aging population subject to varying risks of mortality from different causes.

In this paper, we use the approach proposed by Kashnitsky and Schöley (20) to analyze the spatio-temporal evolution of three groups of causes of death in Brazilian micro-regions (small areas) from 1998 to 2017, by sex, in order to answer the following research question: how does the mortality of a micro-region deviate from the Brazilian average? By doing so, we can visualize deviations from the average Brazilian mortality structure and understand how they evolve over time.

Understanding the spatio-temporal heterogeneity of the causes of death in Brazilian micro-regions is important to develop better public health interventions and to explain the variation and differentials in life expectancy at birth in the country, since regional differences are also reflecting differences in other risk factors, such as population age structure, access to health care, quality of hospital care, and variations in risk behavior. This paper contributes to the literature by focusing on small areas of a diverse country and taking space into consideration as an important variable to understand changes in mortality by causes of death.

## Data and Methods

### Data Source and Level of Analysis

The Brazilian Ministry of Health's database, DATASUS, which is publicly available online (http://www.datasus.gov.br), provides a set of information, such as deaths, causes of death, age, sex, schooling, and race at different geographical levels. Data cleaning and compilation is done at the municipal and state level, and an electronic data file is transferred to the national office every 3 months. The data are available from 1979 and are organized using codes from the International Classification of Diseases Revision (9th from 1980 to 1995 and 10th from 1996 on). For comparability purposes, we use information from 1998 to 2017 (10th Revision). In addition, we decided to work with the most recent data, but also that, at the same time, would provide us a good historical perspective to analyze the spatio-temporal changes. Finally, population by age and sex, at the local level, comes from the Brazilian Censuses (1980, 1991, 2000, and 2010) and intercensal estimates and forecasts from the Brazilian Institute of Geography and Statistics (IBGE).

**Figure 1:**
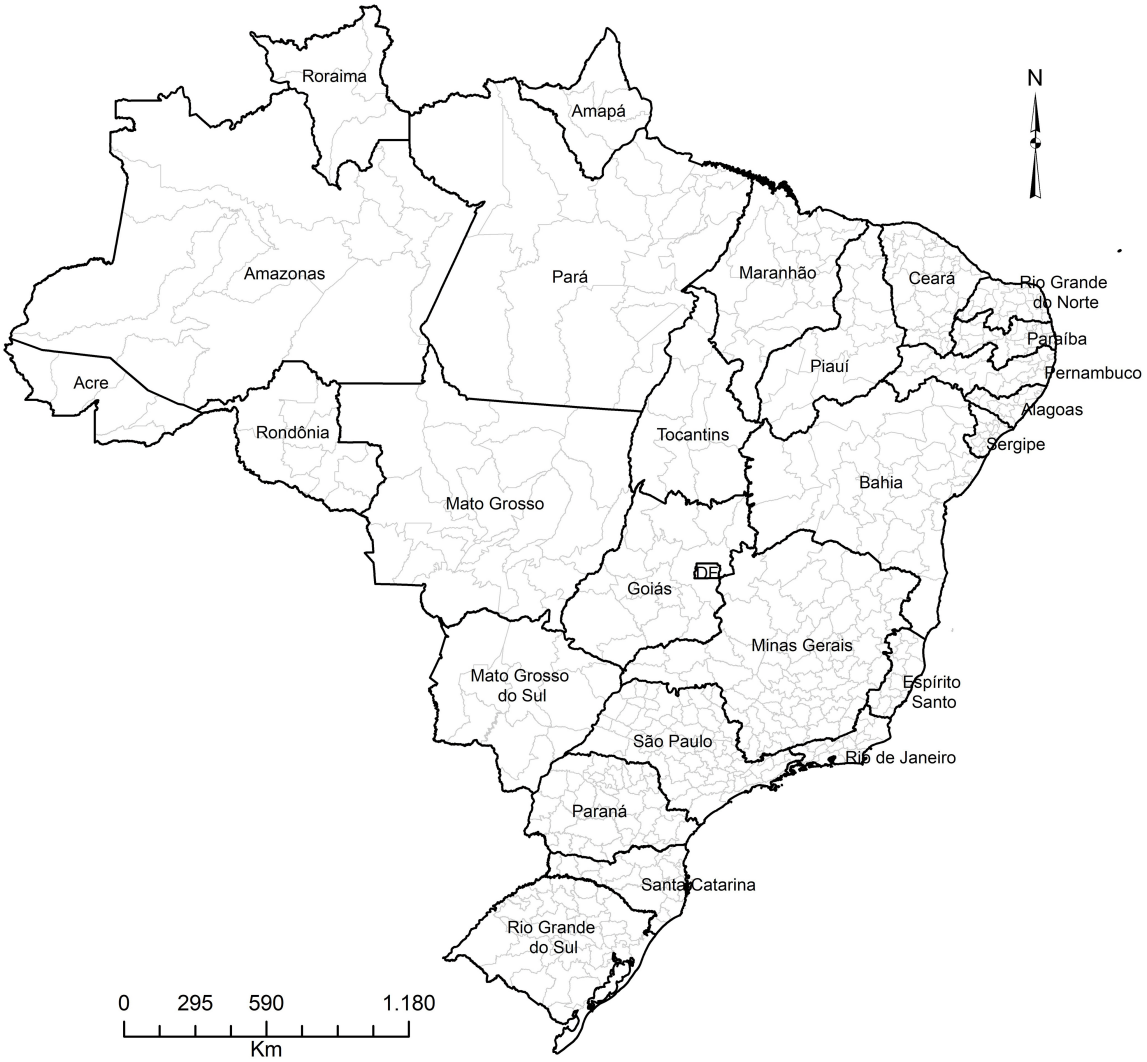
Brazil by states and micro-regions.

The original data is available at the municipality level. The main limitation in using city level data in Brazil is that the number and composition of cities change over time. In 1980, there were 3,974 municipalities and, in 2010, there were 5,565. To avoid problems using this information, the municipalities were aggregated into comparable small areas, using the IBGE definition of geographic micro-regions. These micro-regions do not constitute political or administrative entities, they are statistical constructions aggregated using regional, natural, and socioeconomic similarities (21, 22) and their boundaries are constant throughout the research period. That is, we can follow and study 558 small areas in Brazil (Figure 1) from 1998 to 2017. Recent papers have used this unit of analysis in their studies on mortality in Brazil (16–18).

The second and most important limitation is the quality of mortality data over time and across regions. Historically, states of the North and Northeast had lower coverage and worse quality of death declarations in relation to the states of the Southeast and South (23–26). However, since the 2000s, there has been an impressive improvement in both coverage and quality (25, 26) although, there are still some limitations with quality of registration in Brazil. Several articles demonstrate that the quality of the information has improved, which allows for adequate comparison among the regions (5, 6, 11, 27, 28). Even so, under-registration of death counts by region was corrected using estimates from Lima and Queiroz (25). One shortcoming is that we use the same adjustment factor for all causes of death.

In this paper, causes of death were grouped into three main groups: chronic degenerative diseases - CDDs (cardiovascular diseases and neoplasm), external causes of death (accidents and homicides), and all other causes (a large part of which is infectious diseases). We focus on these specific causes as they represent most deaths observed in the country (during the period studied, 43.10% of deaths were due to CDDs, 12.50% due to external causes, and 44.40% due to all other causes) and have an interesting variation over the life cycle (5, 6, 29). That is, we investigate deaths that are concentrated at younger ages (0–14 years), adult ages (15–59 years), and older ages (over 60 years).

Data from 1998 to 2017 were grouped into four 5-year periods (1998–2002, 2003–2007, 2008–2012, and 2013–2017) to even out any annual fluctuations. In addition, and to avoid impacts of population age structure on the mortality estimates, we standardize mortality rates using the Brazilian population from 2010, similar approach used by Baptista and Queiroz (17, 18). Thus, estimates of causes of death are capturing only the impacts of different mortality levels by cause over time and across space.

Finally, and before turning to the ternary color coding analysis, we presented descriptive statistics for the three main groups of causes of death by region, sex, and period of analysis. In order to compare the differences across years, we apply *t*-test to determine whether mortality differences, by cause and sex, are statistically different from each other. In our case, the numbers of observations are high enough to perform the analysis. In the analysis, we compare the average mortality for each group of cause to the last period of analysis to identify that they are different over time.

### Ternary Color Coding

We used the approach proposed by Kashnitsky and Schöley (20) to investigate the spatio-temporal variation of causes of death in Brazilian micro-regions. The distribution of three groups of causes of death was mapped, rather than showing the proportion of each cause in a separate map, using ternary color coding. According to Schöley (30), this is a technique suitable to the visualization of three part compositions on a surface and that maximizes the amount of information conveyed by colors. It works by expressing the relative shares among three parts, in our case, three groups of causes of death, as the mixture of three primary colors (we define orange as the primary color for CDDs, cyan for external causes of death, and magenta for all other causes). In other words, ternary color coding is designed to visualize proportions of a whole, that is, anything that splits into three non-negative parts that add up to unity. This is perhaps its biggest limitation of the approach, but theoretically, we can input three different variables, and the results will largely be non-sensical. This is because the ternary color coding will convert these variables to proportions that sum to 1, losing all the information about magnitude; that is, the data points 15, 30, 12, and 30, 60, 24, for example, will be converted into the same proportion, 0.26, 0.53, and 0.21, and thus to the same color.

Another limitation occurs when there is unbalanced data, that is, if there is a concentration of observations in one specific variable. In this study, especially for women, there is a concentration of deaths from chronic degenerative diseases (CDDs) and certain other causes. That is, there is little variation with regards to the visual reference point, which is the greypoint that marks perfectly balanced proportions (30). Therefore, to see the internal variation of the data, the point of reference was changed to the average Brazilian mortality structure, thereby, visualizing direction and magnitude of deviations from that average.

## Results

Table 1 presents mean, standard deviation, *t*-test (to test the differences between the means of two group of causes of death), *p*-value, and 95% confidence interval for chronic degenerative diseases, external causes, and other causes across states, by sex and over the periods under analysis. The “Obs” column indicates the number of micro-regions in each state. Overall, we observed an increase in the percentage of deaths from CDDs and external causes in the first three periods, with a stabilization in the most recent one. Females have a higher average mortality rate compared to males with respect to the first group of causes, while for external causes the average is ~3–4 times higher among men. The death rate from CDDs is higher in the South region of the country, while for external causes the Center-West region has the highest averages. In addition, in the analysis, the relationships between the last period (2013–2017) and the others were statistically significant in all situations, indicating the null hypothesis (that the means are equal – using *t*-test) can be rejected.

**Table 1:** Descriptive statistics – chronic degenerative, external causes, and other causes, by sex and period.

	**State**		**Obs**	**(Mean; Std. Dev.) 1998–2002**			**(Mean; Std. Dev.) 2003–2007**			**(Mean; Std. Dev.) 2008–2012**			**(Mean; Std. Dev.) 2013–2017**			**t-statistic**	***p*-value**	**95% CI**
				**Chronic degenerative**	**External causes**	**Other causes**	**Chronic degenerative**	**External causes**	**Other causes**	**Chronic degenerative**	**External causes**	**Other causes**	**Chronic degenerative**	**External causes**	**Other causes**			
Males	Rondônia		8	35.51; 4.37	22.71; 3.46	41.78; 3.28	38.05; 1.97	21.30; 3.68	40.66; 2.46	38.54; 2.52	22.62; 4.10	38.84; 2.94	39.52; 2.50	20.94; 1.92	39.54; 2.77			
	Acre		5	24.04; 7.69	11.10; 4.89	64.86; 11.21	34.20; 3.12	12.08; 3.53	53.71; 4.12	34.02; 2.36	16.63; 1.05	49.35; 3.25	38.53; 4.19	18.67; 1.94	42.79; 2.36			
	Amazonas		13	25.79; 5.87	14.28; 6.25	59.94; 8.22	26.60; 6.26	13.25; 4.93	60.15; 9.82	29.51; 5.92	17.26; 5.23	53.22; 7.95	36.28; 4.80	17.34; 3.95	46.37; 4.53			
	Roraima		4	30.75; 5.92	30.58; 3.31	38.67; 6.00	35.65; 8.09	25.80; 2.92	38.56; 7.20	34.40; 5.17	28.10; 4.57	37.50; 4.19	36.94; 3.94	25.49; 4.00	37.57; 4.36			
	Pará		22	22.70; 7.71	14.03; 7.22	63.27; 11.94	27.96; 5.74	16.83; 8.16	55.21; 10.30	32.70; 4.41	20.73; 7.28	46.57; 7.95	36.05; 3.15	21.44; 5.06	42.52; 4.54			
	Amapá		4	24.33; 9.24	22.04; 5.34	53.63; 7.98	27.71; 4.32	21.73; 3.33	50.56; 5.92	27.39; 8.01	24.73; 4.16	47.88; 4.60	32.50; 5.17	23.22; 5.15	44.27; 1.02			
	Tocantins		8	33.03; 6.05	17.22; 3.49	49.75; 9.26	46.13; 2.17	19.00; 2.05	34.87; 2.50	41.90; 2.67	22.86; 1.65	35.23; 2.64	41.60; 2.63	24.35; 2.18	34.05; 1.98			
	Maranhão		21	22.89; 6.94	11.16; 3.83	65.95; 8.40	34.34; 7.66	13.75; 2.83	51.91; 8.35	41.63; 3.00	19.04; 3.37	39.33; 4.12	41.79; 2.82	19.72; 2.88	38.48; 2.27			
	Piauí		15	31.59; 6.08	11.05; 1.98	57.36; 7.32	38.78; 4.08	14.69; 1.73	46.53; 4.37	44.95; 3.30	19.80; 2.35	35.26; 3.81	44.65; 2.64	18.49; 1.79	36.86; 3.68			
	Ceará		33	31.46; 4.89	17.06; 2.47	51.48; 4.60	37.01; 2.94	19.66; 2.62	43.33; 3.73	39.95; 2.57	23.27; 3.08	36.78; 3.83	39.43; 2.72	23.42; 3.58	37.15; 3.62			
	Rio Grande do Norte		19	26.03; 6.71	16.51; 2.49	57.46; 7.13	37.81; 3.28	18.67; 3.70	43.51; 2.64	41.82; 2.92	20.49; 3.58	37.69; 2.86	39.54; 3.44	21.86; 3.45	38.60; 2.56			
	Paraíba		23	18.15; 5.01	10.22; 3.21	71.63; 7.20	35.18; 5.41	14.46; 2.76	50.36; 5.95	39.74; 2.73	19.05; 2.80	41.21; 3.78	39.21; 2.27	19.46; 1.95	41.33; 2.69			
	Pernambuco		19	27.84; 12.08	19.49; 3.87	52.67; 10.62	35.47; 6.46	20.66; 1.89	43.88; 5.88	39.78; 7.96	20.40; 4.33	39.81; 4.49	38.05; 3.20	19.35; 3.42	42.59; 5.70			
	Alagoas		13	20.97; 5.93	15.56; 2.40	63.47; 6.00	31.61; 3.72	20.30; 1.95	48.09; 3.77	35.00; 3.98	24.62; 2.66	40.39; 3.49	37.12; 2.78	22.40; 1.44	40.48; 2.47			
	Sergipe		13	21.02; 4.76	16.79; 2.97	62.20; 5.93	35.05; 2.58	18.50; 1.99	46.45; 2.37	36.38; 2.01	21.27; 2.44	42.35; 2.33	34.00; 1.80	22.77; 2.57	43.23; 2.26			
	Bahia		32	25.06; 6.40	13.55; 3.23	61.39; 7.80	29.50; 4.84	15.31; 3.36	55.20; 6.10	32.58; 4.14	20.59; 3.46	46.82; 4.90	32.99; 4.07	21.00; 2.84	46.01; 4.60			
	Minas Gerais		66	36.93; 7.42	10.30; 2.13	52.77; 7.97	39.67; 7.34	12.11; 2.68	48.22; 6.95	38.47; 5.36	15.22; 2.80	46.31; 5.35	38.24; 4.20	16.74; 3.22	45.03; 3.70			
	Espírito Santo		13	37.04; 4.20	17.83; 3.05	45.13; 5.45	45.89; 3.88	19.53; 2.82	34.58; 3.66	43.61; 2.43	24.51; 2.77	31.88; 1.95	42.76; 2.92	23.82; 2.59	33.42; 2.12			
	Rio de Janeiro		18	43.67; 4.70	16.40; 4.21	39.93; 2.90	44.96; 4.99	16.07; 4.14	38.97; 3.02	43.69; 3.38	16.68; 3.24	39.64; 2.04	42.70; 3.58	17.01; 3.08	40.29; 2.04			
	São Paulo		63	42.34; 4.68	14.08; 3.06	43.58; 5.14	43.18; 4.78	12.77; 1.89	44.05; 4.84	41.80; 4.49	12.96; 1.69	45.24; 4.56	42.58; 4.16	12.42; 1.51	45.00; 4.22			
	Paraná		39	47.36; 4.70	14.37; 2.23	38.27; 4.10	48.01; 3.62	15.49; 2.72	36.50; 3.57	44.27; 3.51	18.67; 2.68	37.05; 3.36	43.70; 2.53	18.55; 2.30	37.75; 2.72			
	Santa Catarina		20	44.02; 3.76	13.93; 1.64	42.05; 3.53	45.99; 3.97	14.87; 1.51	39.14; 3.51	46.02; 3.57	16.32; 2.14	37.66; 3.26	45.74; 3.38	16.41; 1.95	37.86; 3.21			
	Rio Grande do Sul		35	48.59; 2.80	12.77; 1.90	38.64; 2.96	48.89; 2.57	13.02; 2.05	38.08; 2.37	47.29; 2.76	14.77; 2.35	37.95; 1.95	45.71; 3.01	15.84; 2.45	38.44; 2.38			
	Mato Grosso do Sul		11	43.42; 3.80	17.46; 2.26	39.12; 4.86	47.56; 2.39	17.77; 1.89	34.67; 3.53	44.42; 1.85	19.97; 3.16	35.61; 3.03	43.74; 1.99	18.63; 2.73	37.64; 3.17			
	Mato Grosso		22	40.80; 3.78	21.97; 3.95	37.22; 5.11	42.79; 3.47	20.36; 2.97	36.86; 3.37	38.50; 2.76	22.89; 2.66	38.61; 4.10	35.83; 3.42	23.05; 2.64	41.12; 3.77			
	Goiás		18	37.35; 3.99	17.69; 2.30	44.97; 4.14	40.13; 3.91	16.88; 2.11	42.99; 3.58	39.00; 1.96	19.62; 2.48	41.39; 2.27	38.73; 1.84	22.49; 2.29	38.78; 1.76			
	Distrito Federal		1	46.35; NA	17.10; NA	36.56; NA	49.50; NA	16.19; NA	34.30; NA	45.91; NA	19.69; NA	34.40; NA	45.23; NA	18.12; NA	36.65; NA			
	Overall		558	34.77; 10.87	14.71; 4.71	50.53; 11.92	39.57; 7.89	15.80; 4.28	44.63; 8.28	40.10; 5.89	18.60; 4.69	41.29; 6.20	40.05; 4.89	18.90; 4.39	41.05; 4.87			
	1998–2002 vs. 2013–2017																	
		Chronic degenerative														−13.59	<2e−16 ^***^	[−6.05–−4.52]
		External causes														−22.03	<2e−16 ^***^	[−4.56–−3.82]
		Other causes														19.51	1.33e−12 ^***^	[8.52–10.43]
	2003–2007 vs. 2013–2017																	
		Chronic degenerative														−1.98	<2e−16 ^***^	[−0.97–−0.004]
		External causes														−21.76	<2e−16 ^***^	[−3.38–−2.82]
		Other causes														12.49	<2e−16 ^***^	[3.02–4.15]
	2008–2012 vs. 2013–2017																	
		Chronic degenerative														0.31	<2e−16 ^***^	[−0.25–0.35]
		External causes														−2.97	<2e−16 ^***^	[−0.49–0.10]
		Other causes														1.55	<2e−16 ^***^	[−0.07–0.56]
Females	Rondônia		8	44.17; 2.87	5.43; 1.21	50.40; 2.36	47.02; 2.98	5.43; 0.71	47.54; 2.46	46.61; 2.76	6.31; 0.86	47.09; 3.13	46.36; 3.05	5.75; 0.73	47.89; 3.06			
	Acre		5	28.35; 7.07	3.63; 1.82	68.02; 8.84	40.12; 1.04	3.13; 1.01	56.75; 1.54	40.20; 2.15	4.40; 0.69	55.41; 2.49	44.93; 3.35	4.21; 0.38	50.86; 3.26			
	Amazonas		13	29.50; 9.14	3.65; 3.20	66.85; 10.78	31.41; 6.51	3.24; 1.19	65.35; 6.19	35.16; 6.43	4.39; 1.98	60.45; 6.80	42.71; 6.30	3.60; 1.71	53.69; 5.01			
	Roraima		4	35.38; 9.34	7.43; 1.72	57.19; 9.04	42.62; 8.73	5.82; 0.68	51.56; 8.75	46.53; 8.57	7.87; 1.09	45.60; 8.03	39.97; 4.47	8.08; 1.61	51.95; 3.55			
	Pará		22	27.25; 10.14	3.24; 1.70	69.50; 11.05	34.99; 8.91	3.70; 1.71	61.30; 10.0	41.45; 5.62	4.33; 2.30	54.22; 6.50	44.05; 3.59	4.51; 1.04	51.45; 3.62			
	Amapá		4	28.51; 12.71	2.74; 2.04	68.76; 13.28	31.31; 9.78	5.71; 2.28	62.97; 8.32	37.13; 4.28	6.58; 1.75	56.29; 4.54	37.91; 7.74	5.02; 2.13	57.07; 8.21			
	Tocantins		8	40.70; 7.91	4.96; 1.27	54.34; 8.99	56.25; 2.52	4.86; 0.65	38.89; 2.67	51.86; 2.03	6.59; 0.87	41.55; 2.38	50.84; 3.64	6.78; 1.12	42.38; 2.85			
	Maranhão		21	25.82; 8.33	3.09; 1.09	71.09; 8.97	40.36; 8.57	3.63; 0.71	56.00; 8.76	51.54; 3.29	4.47; 1.04	43.99; 3.60	50.81; 2.25	4.78; 0.86	44.41; 2.18			
	Piauí		15	33.18; 7.92	2.97; 0.91	63.85; 8.14	46.10; 5.78	3.47; 0.57	50.42; 5.73	53.50; 4.01	5.18; 0.97	41.31; 4.18	52.75; 4.46	4.90; 0.72	42.35; 4.72			
	Ceará		33	39.11; 5.43	3.92; 0.78	56.97; 5.44	47.24; 4.06	3.89; 0.59	48.87; 4.20	52.73; 3.70	4.67; 0.92	42.59; 3.82	50.28; 3.51	4.71; 0.90	45.02; 3.79			
	Rio Grande do Norte		19	30.57; 7.72	3.75; 0.73	65.68; 7.74	45.80; 3.11	3.86; 0.53	50.34; 3.13	52.40; 3.13	4.52; 0.86	43.09; 3.14	48.85; 2.66	4.54; 0.88	46.61; 2.58			
	Paraíba		23	21.25; 5.80	2.12; 0.68	76.63; 6.04	42.27; 6.93	2.90; 0.62	54.84; 7.10	49.70; 3.49	3.92; 0.84	46.38; 3.52	47.85; 2.65	4.02; 0.75	48.14; 2.98			
	Pernambuco		19	32.52; 10.70	3.45; 1.06	64.03; 10.97	42.70; 12.74	3.60; 1.09	53.70; 13.40	50.86; 8.99	4.41; 1.29	44.73; 8.10	49.68; 12.31	4.08; 1.22	46.24; 11.39			
	Alagoas		13	25.56; 6.91	2.94; 0.56	71.50; 6.93	40.35; 4.69	3.62; 0.63	56.03; 4.70	46.16; 4.14	4.28; 1.03	49.56; 3.96	47.86; 2.87	4.07; 0.66	48.07; 2.74			
	Sergipe		13	26.14; 6.95	3.57; 0.86	70.30; 7.08	45.57; 2.63	3.86; 0.75	50.57; 2.81	48.94; 2.72	5.19; 0.84	45.87; 2.60	45.82; 2.52	5.68; 1.11	48.49; 2.57			
	Bahia		32	28.46; 8.66	3.55; 0.92	67.99; 9.25	35.96; 6.17	3.88; 0.84	60.16; 6.45	41.50; 5.18	4.89; 0.79	53.60; 5.46	41.28; 4.55	4.90; 0.81	53.82; 4.62			
	Minas Gerais		66	42.38; 8.11	3.38; 0.79	54.25; 8.30	44.97; 7.27	3.62; 0.80	51.41; 7.24	44.91; 5.12	4.76; 0.84	50.33; 5.16	44.10; 3.62	5.09; 0.89	50.81; 3.74			
	Espírito Santo		13	42.26; 5.56	5.02; 0.83	52.72; 5.74	52.43; 4.50	5.83; 0.93	41.74; 4.28	52.66; 2.31	7.47; 0.86	39.87; 2.68	49.16; 1.76	8.04; 1.02	42.81; 1.88			
	Rio de Janeiro		18	50.55; 3.78	4.68; 1.03	44.77; 3.39	50.47; 3.60	4.55; 0.97	44.98; 3.51	48.57; 2.98	5.61; 1.35	45.82; 2.92	46.47; 3.33	5.50; 0.85	48.04; 3.13			
	São Paulo		63	49.78; 5.08	4.24; 0.69	45.98; 5.08	48.81; 4.82	3.93; 0.69	47.26; 4.93	46.78; 4.55	4.25; 0.75	48.96; 4.61	45.81; 4.29	4.43; 0.97	49.76; 4.17			
	Paraná		39	54.50; 4.46	4.11; 0.85	41.40; 4.67	54.57; 4.03	4.36; 0.82	41.07; 4.20	51.57; 3.26	5.43; 1.09	43.00; 3.30	50.04; 2.85	5.59; 1.02	44.36; 2.88			
	Santa Catarina		20	49.92; 4.35	4.59; 0.82	45.49; 4.42	51.33; 4.19	4.65; 0.72	44.02; 4.12	51.22; 3.62	5.28; 1.15	43.50; 3.65	50.59; 3.54	5.79; 1.12	43.61; 3.53			
	Rio Grande do Sul		35	56.36; 3.20	4.05; 0.71	39.59; 3.26	56.23; 2.60	4.10; 0.80	39.67; 2.70	53.40; 2.43	4.89; 1.17	41.71; 2.33	51.26; 2.75	5.43; 1.31	43.30; 2.79			
	Mato Grosso do Sul		11	51.83; 4.18	5.22; 0.70	42.95; 4.46	55.05; 2.85	5.27; 1.22	39.68; 3.14	51.00; 2.42	6.74; 1.27	42.25; 2.71	48.80; 1.94	6.86; 1.61	44.34; 2.81			
	Mato Grosso		22	51.14; 4.96	6.19; 1.38	42.67; 5.35	51.56; 2.63	5.75; 1.57	42.69; 2.80	47.31; 3.17	6.75; 1.36	45.94; 3.68	44.27; 2.81	6.49; 1.01	49.23; 3.31			
	Goiás		18	45.74; 4.64	5.33; 0.50	48.94; 4.89	47.88; 3.82	5.02; 0.63	47.10; 3.83	46.59; 2.30	6.15; 0.68	47.25; 2.41	46.21; 2.49	7.22; 0.85	46.57; 2.77			
	Distrito Federal		1	55.06; NA	5.11; NA	39.84; NA	55.21; NA	5.57; NA	39.22; NA	53.70; NA	6.28; NA	40.02; NA	51.96; NA	6.22; NA	41.82; NA			
	Overall		558	40.72; 12.62	3.96; 1.36	55.32; 13.27	46.47; 8.51	4.10; 1.12	49.43; 8.89	48.13; 6.02	5.03; 1.38	46.84; 6.24	47.12; 5.17	5.17; 1.38	47.71; 5.25			
	1998–2002 vs. 2013–2017																	
		Chronic degenerative														−12.01	5.13e−07 ^***^	[−7.44–−5.35]
		External causes														−20.65	<2e−16 ^***^	[−1.32–−1.09]
		Other causes														13.97	4.08e−11 ^***^	[6.54–8.67]
	2003–2007 vs. 2013–2017																	
		Chronic degenerative														−1.85	<2e−16 ^***^	[−1.32–0.04]
		External causes														−22.60	<2e−16 ^***^	[−1.17–−0.98]
		Other causes														4.96	<2e−16 ^***^	[1.04–2.40]
	2008–2012 vs. 2013–2017																	
		Chronic degenerative														6.24	<2e−16 ^***^	[0.69–1.33]
		External causes														−3.07	<2e−16 ^***^	[−0.22–−0.05]
		Other causes														−5.34	<2e−16 ^***^	[−1.20–−0.55]

*Signif. codes*:

* *p ≤ 0.05*;

** *p ≤ 0.01*;

*** *p ≤ 0.001*.

**Figure 2:**
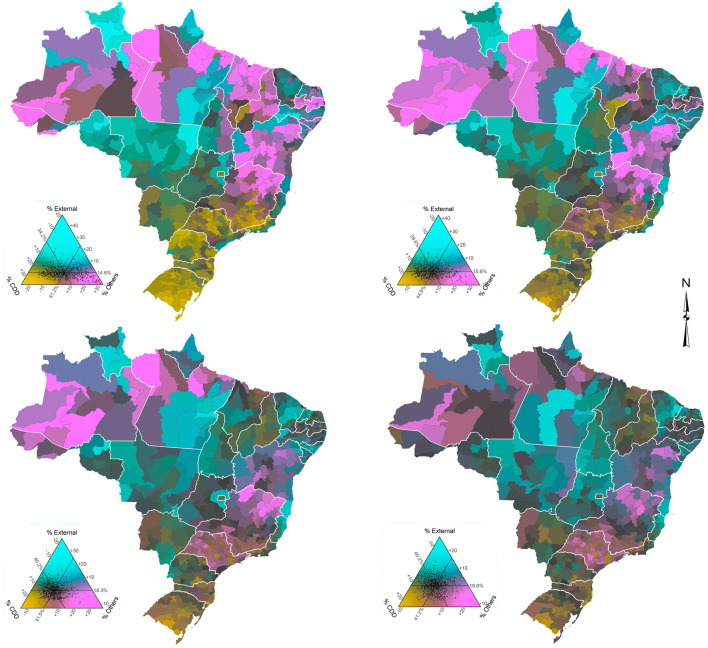
Distribution of groups of causes of death by micro-regions, males, Brazil - time series 1998–2002 (top left), 2003–2007 (top right), 2008–2012 (bottom left), and 2013–2017 (bottom right).

**Figure 3:**
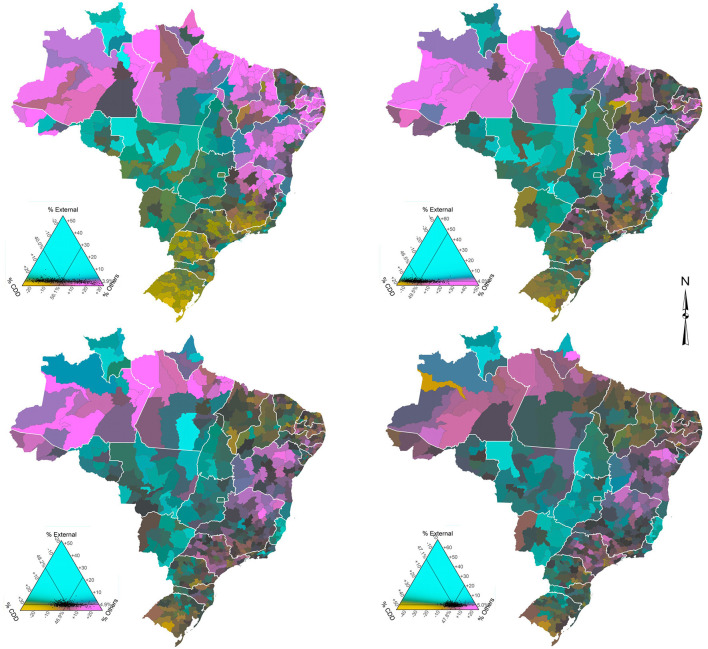
Distribution of groups of causes of death by micro-regions, females, Brazil - time series 1998–2002 (top left), 2003–2007 (top right), 2008–2012 (bottom left), and 2013–2017 (bottom right).

Figure 2 (males) and Figure 3 (females) show the proportional distribution of groups of causes of death across Brazilian micro-regions in the four 5-year periods studied. Before presenting the main findings, we show an example to interpret the ternary color coding. For this, we use males (Figure 2) for the period 1998–2002 (top left) as an example. Each point within the triangle represents a micro-region. The reading on the percentage observed in each group of causes of death and micro-region occurs in a clockwise direction. Therefore, in this study, the percentage of CDDs can be read on the left side of the triangle, external causes on the right side, and all other causes at the bottom. Taking the visual reference point (point of intersection of the three lines within the triangle) as an example, which is the average Brazilian mortality structure, the observed percentages are 34.2, 14.6, and 51.2 for CDDs, external causes, and all other causes, respectively. The darker the color of a micro-region, the higher the share of deaths observed for that group of causes. On the other hand, the grayer a micro-region is, the more balanced the three proportions are.

Regarding the overall results, they indicate that, when comparing males and females, the percentage of deaths due to the groups of CDDs, and other causes is higher for females in all periods and states, while the percentage of deaths due to external causes is higher for males. In addition, results confirm that the ternary compositions are much clearer and more spread out for men, since the data are more balanced.

External causes represent the largest percentage difference between the sexes. Only the state of São Paulo, the most developed state in the country, has a percentage difference of <10 in all periods. The state of Rio Grande do Sul slightly exceeds this percentage in the last period of analysis. At the other end, the percentage difference in Roraima is ~20% greater for males, although, it shows a slight decline in the period 2013–2017. For males, external causes of death increased very rapidly since the 1980's, having negative impacts on the variation and evolution of life expectancy in Brazil and increasing the gender gap. In addition, there are important changes in the spatial distribution over time.

As for the spatio-temporal variation, and taking into account the visual reference point, Figures 2, 3 show that the average Brazilian mortality structure showed very similar changes of intensity for both sexes over the years, although the changes were quite different proportionally. Roughly speaking, CDDs increased significantly from 1998–2002 to 2003–2007 and then “stabilized” (proportionately) in the following years. Meanwhile, external causes remained constant in the first two periods and rapidly increased from 2003–2007 to 2008–2012, with the last period being similar to the previous one. Finally, other causes showed a significant drop between the first two periods and a less intense drop between 2003–2007 and 2008–2012, with the last period (2013–2017) also stable in relation to the previous one.

A more specific analysis of the spatio-temporal variation by sex shows that for males (Figure 2), the micro-regions of the North and Northeast, less developed and poorest regions of the country, have shown a reduction in the proportion of deaths from other causes over the years, particularly the micro-regions of the states of Acre, Amazonas, and Pará (North) and Maranhão, Piauí, Paraíba, Alagoas, Sergipe, and Bahia (Northeast), while an increase in the proportion of deaths from CDDs is observed. This combination reflects a narrowing gap between these regions and the average Brazilian mortality structure. In the Center-West, Southeast, and South regions, the proportion of CDDs increased between 1998–2002 and 2003–2007 in all states but declined in the following years. Meanwhile, other diseases made the reverse movement. In summary, the mortality structure observed in the states that are closest to the average Brazilian mortality structure for men are Tocantins (North) and Ceará (Northeast) - 1998–2002; Piauí (Northeast) - 2003–2007; Maranhão and Paraíba (Northeast) and Goiás (Center-West) - 2008–2012; and Maranhão and Paraíba (Northeast) - 2013–2017.

Regarding females (Figure 3), the first observation concerns the low percentage and small temporal variation in deaths from external causes registered in most micro-regions, which demonstrates how highly unbalanced the data are. This also makes the averages of micro-regions closer to the national average. The states with the highest percentages of deaths from external causes are Roraima (North), Espírito Santo (Southeast), and Mato Grosso (Center-West), while the lowest percentage is observed in the state of Paraíba (Northeast). As the weight of external causes is much lower in the mortality of women compared to men, the percentages of CDDs and other causes acquire additional importance, although, the spatio-temporal configuration is often like those observed in men. The Northern and Northeastern micro-regions showed a significant increase in the proportion of deaths from CDDs between 1998 and 2012 (first three periods), while the same intensity was observed in the reduction of deaths from other causes in the period. However, in the last period (2013–2017), the process was reversed in most states in these regions, although, to a much lesser intensity. That is, there was an increase in the proportion of deaths from other diseases and a decrease in the proportion of deaths from CDDs. In the micro-regions of the Center-West, Southeast, and South, in general, a small increase in the proportion of deaths from CDDs and a decrease in deaths from other causes between 1998–2002 and 2003–2007 was observed. However, in the following years the movement was reversed. Specifically, the state of Minas Gerais (Southeast) draws attention, particularly the micro-regions of the north/northeast of the state, where there was a drop in the proportion of deaths from other causes and a significant increase in chronic degenerative diseases between 1998–2002 and 2003–2007. In addition, the states of Rio de Janeiro and São Paulo (Southeast) and Rio Grande do Sul (South) were the only ones in these regions that observed a drop in the proportion of deaths from CDDs and an increase in the proportion of deaths from other causes throughout the period. We can also observe that, over the years, the mortality structure of women in micro-regions has converged to the average Brazilian mortality structure, a slightly different situation from what we see for men. In summary, the mortality structure observed in the states that are closest to the average Brazilian mortality structure for women are Ceará (Northeast) - 1998–2002; Piauí, Ceará, Rio Grande do Norte, and Sergipe (Northeast) - 2003–2007; Sergipe (Northeast) and Rio de Janeiro (Southeast) - 2008–2012; and Rondônia (North), Rio Grande do Norte, Paraíba and Alagoas (Northeast) and Rio de Janeiro (Southeast) -2013–2017.

## Discussion

In recent years, few studies have investigated the variation of mortality by causes of death in Brazil taking both time and spatial factors into consideration, which makes us understand that further studies in this area are needed. Borges (5) examines health and mortality transitions across regions in Brazil and shows that there is a regional variation in the improvement of mortality from chronic degenerative diseases: faster declines were observed in the Southern regions of the country, but the persistence of high levels of mortality due to infectious diseases remained in the northern parts of the country. Borges (5) also showed the negative impact of external causes of death on the variation in life expectancy across the country. França et al. (6), based on estimates of the GBD study, investigate the trends in mortality and causes of death across states from 1990 to 2017. As in previous studies, they show a rapid increase in life expectancy and, similar to Borges (5), a variation in the causes of death across regions and over time. However, both studies focus on larger areas and miss important variations within states that are also marked by high income and social inequalities that might affect both the intensity and distribution of different causes of death. The results for regions and states do not represent the differences observed within each area. For instance, in the state of Minas Gerais the results indicate different regional patterns of mortality by cause. Therefore, understanding the spatial pattern of mortality by cause and sex, especially in small areas, becomes extremely relevant for public health policies and the well-being population, as these policies must be elaborated and spatially focused to serve a population that is heterogeneous in its composition, and that, consequently, presents specific and varied demands.

In the last two decades, the distribution of analyzed causes of deaths changed in most micro-regions. In parallel with spatio-temporal variations, there is a significant gender gap in Brazil in relation to mortality. This can be seen in a higher life expectancy at birth for women, which is 7.1 years higher than it is for men (2). Regionally, there are important differences in the trends in causes of deaths and mortality patterns for males and females.

Regarding mortality from external causes, we observed that the impact is, on average, four times greater for males. The results showed that the mortality of males due to external causes has spread to several micro-regions in the North and Northeast regions, while for females the pattern is less clear. This pattern is in line with what is observed in other studies, although, most of those studies are focused on a specific group or category of mortality from external causes (5, 19, 31–36). In the states of the South and Southeast regions, mortality from homicides increased between 1980 and 2000, while in Northern and Northeastern states, violent deaths rose more markedly after 2000 (31–34). At the state level, between 2001 and 2011 in all Northeastern states (except Pernambuco), homicide rates increased (31). Meanwhile, data show that in six states in the North and Northeast regions, homicide mortality more than doubled between 2005 and 2015 (33). On the other hand, the same study indicates homicide mortality diminution in all Southeastern states. Traffic accidents (TA) represent the other main component of mortality from external causes (32, 35, 36). According to Morais-Neto et al. (37), between 2000 and 2010, mortality from traffic accidents increased substantially in Brazil. However, there are clear regional differences in the TA mortality trends (37, 38). Mortality from motorcycle accidents, for example, has increased significantly in North, Northeast, and Center-West regions in recent decades (19, 37, 39). Pinheiro et al. (40) found that, between 2005 and 2015, several municipalities in the western portion of Paraná, passing through the Center-West and Northeast regions, presented a high concentration of high motorcycle mortality rates among women. To some extent, this spatial pattern can also be observed in our findings.

In the last decades, communicable disease mortality in Brazil has been steadily decreasing (6, 7). In states such as Amazonas, Rondônia, Pernambuco, Minas Gerais, Goiás, and Distrito Federal, as well as in all states in the southern region, the disability-adjusted life year (DALY) of neglected tropical diseases rates (infectious and parasitic diseases) fell more than 40% between 1990 and 2016 (7). Leite et al. (41) observed that in 2008 infectious and parasitic diseases represented a large share of DALY in North (18.3%) and Northeast (15.7%) regions. The DALY proportion represented by that group of causes of death in the same regions was 32.7 and 30.1% in 1998, respectively (42). The maps presented seem to agree with these estimates, as other causes of death, such as infectious and parasitic diseases, were still relevant in these regions, even in the last period, although to a lesser extent.

Finally, chronic degenerative diseases, more specifically CVDs and neoplasm, represent ~47% of all deaths that occur in Brazil (43). In line with other studies, the results show a higher proportional increase in deaths from CDDs in the micro-regions of the North and Northeast, and that men have higher mortality than women (6, 44). Baptista and Queiroz (17, 18) show that less developed regions, such as the North and Northeast, have experienced a more rapid increase in mortality from cardiovascular diseases in recent years. Compared to neoplasms, its contribution to overall mortality has increased steadily from 1991 to 2010, for both males and females (14). The change is closely related to changes in population age structure. Overall, neoplasms are the second major cause of death in Brazil. In 1990, the highest level of mortality due to neoplasm was concentrated in the South region of the country, but a spread in mortality from this cause to all regions was observed by 2010. In 2010, almost all regions in the South and Southeast of the country had very high mortality rates from this particular cause, as did some areas of the Northeast (45). There is variation across regions in the level and the timing of mortality change from causes that directly influence the gender gap. A decline in female mortality from CVDs started first and decreased at higher levels in the states of the Southeast and South regions (5, 13). Throughout 1998-2017, in comparison with males, there was a clear reduction in the number of Southern and Southeastern micro-regions with above average CDD mortality for females (15). In recent years, the decline in CVDs has been related to a decline in risk factors associated with the individuals targeted by policies, such as tobacco control and programs to improve preventive care for high blood pressure (6, 13). However, the quality of hospital care plays an important role in the declining trend and there are large regional differences in assistance quality (13).

The diverse color-coded figures across space and time show the different stages of the epidemiological transition across different regions of the country. Epidemiological transition is not happening uniformly across the country and the prevalence of diseases varies substantially. For instance, for males in 2013-2017, we observe higher levels of external causes of death (homicides and accidents) in the interior and coastal areas of the Northeast. For females, external causes of death are concentrated in the Southeast region, more specifically in micro regions in the state of Rio de Janeiro. This implies that the Brazilian health care system needs to elaborate specific policies and interventions for each area, taking into consideration the demographic dimension, as well as the specificities of the health transition. The previous results indicate that a national health policy, or even a state level one, is not the best alternative to cover the public health demand in the country.

## Conclusion

In this paper, our contribution was to study the spatio-temporal evolution of the main causes of death across small areas and over time in Brazil. This is helpful in understanding the dynamics of the health transition in Brazil in a recent period of time and it can have a positive impact on public health policies. Results are in line with other studies that focus on states or major regions of the country, but we provide results for small regions over a relatively long period of time. Mortality levels by different diseases are related to population age structure, health conditions, institutional factors, the environment and the socioeconomic situation to which the population is exposed. Brazil is marked by large socioeconomic differences with strong relations to regional diversities (46). These relations offer possibilities for targeted public interventions, but at the same time present challenges that a country of continental dimensions cannot avoid. Brazilian health-system planning requires an understanding of the absolute burden of the main causes of deaths and the effect of changes in population age structure.

Regional trends in early mortality by chronic and degenerative diseases should be carefully analyzed, so as to better define strategies to address the realities in different regions of the country. Results also showed the transition over time regarding external causes of deaths and the role they play in explaining the gap in male and female mortality. In the context of the Covid-19 pandemic, our results also shed some light on which regions might be more vulnerable to the impacts of the pandemic given the recent trends in causes of deaths, since several studies showed that individuals with certain comorbidities are more susceptible to more severe cases of the disease.

Finally, the analysis highlights the importance of combining spatial and demographic analysis using data visualization approaches. The map color scheme allows us to study and observe changes in mortality from various causes in the same figure instead of looking at each cause in a different figure. In addition, it combines spatial and temporal analysis, which makes it easier to observe important and interesting trends.

## Data Availability Statement

The original contributions presented in the study are included in the article/supplementary material, further inquiries can be directed to the corresponding author/s.

## Author Contributions

EB, BQ, and PP contributed to the design and analysis of the study, the writing of the manuscript, and revising the paper. EB gathered the data and prepared the first round of analysis. BQ and PP revised the estimates and analysis. All authors read and approved the final manuscript.

## Conflict of Interest

The authors declare that the research was conducted in the absence of any commercial or financial relationships that could be construed as a potential conflict of interest.
